# Effect of glucose on medium chain triglyceride induced ketosis in healthy adults in a randomized, double-blind, controlled study

**DOI:** 10.1038/s41598-026-47702-4

**Published:** 2026-04-09

**Authors:** Marius Frenser, Manfred Fobker, Renata Antonina Feuerborn, Thorsten Marquardt, Tobias Fischer

**Affiliations:** 1 Center for Nutrition and Therapy (NuT), FH Muenster, University of Applied Sciences Muenster, 48149 Muenster, Germany; 2 Centre of Laboratory Medicine, University Hospital Muenster, 48149 Muenster, Germany; 3 Department of General Pediatrics, Metabolic Diseases, University Hospital Muenster, 48149 Muenster, Germany

**Keywords:** Ketone bodies, Caprylic acid (C8; C8:0), Tricaprylin, Medium-chain triglycerides (MCT), Ketogenic diet, Glucose, Diseases, Endocrinology, Medical research, Physiology

## Abstract

Although medium-chain triglycerides (MCT) stimulate and carbohydrates inhibit ketone body synthesis, their quantitative interaction in humans is not well defined. This randomised, controlled, double-blind crossover study investigated how increasing glucose doses affects C8-MCT-induced ketone body synthesis after overnight fasting. Eleven healthy young woman (22.5 ± 1.9 years) received a constant dose of tricaprylin (99% C8; 0.2 g/kg bodyweight) combined with increasing glucose doses (0.2–0.6 g/kg bodyweight). In additional interventions, both C8-MCT and glucose were increased in parallel. Plasma β-hydroxybutyrate (βHB), glucose, and insulin were measured for up to 300 min post-dose. Indirect calorimetry in a subset and side effects were monitored. C8-MCT significantly increased βHB concentrations at low and medium glucose doses compared with control, but not at highest glucose dose. When both substrates were increased equally (1:1 ratio), ketone body synthesis increased. βHB was negative correlated with increasing glucose dosing at constant C8-MCT dosing, while parallel increases in both substrates showed a positive moderate correlation between βHB and C8-MCT doses. No dose-dependent side effects occurred. Overall, ketone body synthesis declines with rising glucose intake. It remains unclear whether a C8-MCT: glucose ratio of 1:3 represents a metabolic cut-off or whether glucose intake becomes excessive due to linear effects.

*Trial registration* This study was prospectively registered in the German Clinical Trials Register (DRKS ID DRKS00035373 https//www.bfarm.de/EN/BfArM/Tasks/GermanClinicalTrialsRegister/_node.html).

**Supplementary Information:**

The online version contains supplementary material available at 10.1038/s41598-026-47702-4.

## Introduction

The implementation of ketogenic diets is often associated with practical challenges and high dropout and non-compliance rates^1,2^, which reduce treatment success^3^. Increasing the carbohydrate (CH) intake within an medium-chain triglyceride (MCT)-based ketogenic diet (MCTKD) could mitigate this and improve compliance^4^, thus increasing the suitability of ketogenic diets for everyday use. MCT are composed of medium-chain fatty acids (MCFA) with 6–12 carbon atoms, which are esterified with glycerol^5,6^. While the majority of plant-based foods are rich in long-chain triglycerides (LCT), palm kernel oil and coconut oil in particular contain approximately 50% MCFA in their total fatty acid content. The primary constituent of coconut and palm kernel oil is C12, with smaller quantities of fatty acids with shorter chain lengths^7,8^. Other natural sources include milk fat (4–12% MCFA of total fatty acids), butter (8%) and cheese (7.4%), but these have considerably lower concentrations of MCFA^6,8^. Conventional MCT oils available on the market are mostly C8/C10 mixtures but pure C8 oils are also available^7,9,10^. MCT are more ketogenic than LCT^11–14^, with C8 being attributed the strongest ketogenic effect, with approximately three and six times higher ketogenic activity compared to C10 and C12, respectively^13^. C6 oils are not available on the market, but in animal studies they are even more ketogenic than C8 ^15^. MCT metabolism differs notably from that of LCT. After ingestion, MCT are hydrolysed by lipases and the resulting MCFA are absorbed by enterocytes, where they can diffuse across cell membranes without requiring specific transporters. They are transported predominantly unbound via the portal vein to the liver^5,16–18^. MCFA can enter the mitochondrial matrix independently of the carnitine shuttle. Within the mitochondria, MCFA undergo beta oxidation to yield acetyl-CoA. When there is an excess of acetyl-CoA, the ketone bodies acetoacetate (AcAc), β-hydroxybutyrate (βHB) and acetone are formed^17,19,20^. βHB is particularly important for the human organism’s energy supply^21,22^. Despite the presence of β-ketoacyl-CoA transferase, the liver cannot utilise ketone bodies, which are therefore transported to other tissues such as muscles and brain for energy production^7^. Conversely, oral CH induce insulin secretion^23^, which suppresses lipolysis and fatty acid oxidation and reduces hepatic ketone body synthesis^24–26^. A meta-analysis of the influence of CH in combination with C8 on ketone body synthesis suggested that a higher C8 intake of ⌀ 18.6 ± 0.9 g C8 compared to ⌀ 11.4 ± 1.0 g C8 did not lead to significantly higher ketone body synthesis despite low CH intake (53.3 ± 18.8 g instead of 61.1 ± 6.1 g)^27^. However, studys were inconsistent due to differences in caffeine intake and different types of dispersion, which can have an influence on ketone body synthesis^28–30^. In addition, it was found that simultaneous CH and MCT intake leads to lower ketone body levels than MCT intake alone^27^. In an intervention study by Heidt et al. (2023), this effect could not be demonstrated^12^. None of the studies systematically examined the interaction between different carbohydrate and MCT dosages in a controlled, dose-dependent manner. Consequently, the quantitative relationship between carbohydrate intake and MCT-induced ketone body synthesis remains insufficiently characterized. The current study primary aimed to investigate the effect of increasing glucose doses on MCT-inducted ketone body synthesis in order to identify a possible cut-off point and secondary to examine linearity of increasing MCT and glucose doses. Due to the superior ketogenic effect of C8, C8-based MCT was used. Relative contribution of macronutrients to energy expenditure was additionally secondary assessed by indirect calorimetry in a subset.

## Materials and methods

### Study sample

Thirteen healthy participants were included in the study. Participants of all sexes (female / male / all genders) were recruited between the ages of 18 and 35 years with normal weight (BMI 18.5–25.0) and without any known metabolic, serious, chronic or acute diseases or diagnosed eating disorders. Exclusion criteria were the use of dietary supplements that affect lipid metabolism, medication, pregnancy and breastfeeding, mental or linguistic impairments, a low-carbohydrate or other diet with a permanent reduction in CH intake (< 50% of energy intake), whole blood and blood plasma donations in the last 12 or 4 weeks prior to the start of the study, and intolerance to sweeteners. Ethical approval was obtained from the responsible ethics committee of the Westphalia-Lippe Medical Association prior to the start of the study (approval number: 2024-503-f-S). All participants provided written informed consent. The study design and conduct comply with the requirements of the Declaration of Helsinki (2013) and were prospectively registered as a clinical trial in the DRKS (DRKS ID: DRKS00035373; registration date: 31.10.2024).

### Test ingredients

The intervention drinks contained 250 ml water, 15 µl flavouring (Givaudan, Netherlands/Germany or Pure Flavour, Germany), C8 oil (Ketosource, England; 99% C8) and anhydrous glucose (Thermo Fisher Scientific, Belgium; 99,5% glucose) with doses depending on body weight (bw). Sucralose (Givaudan, Switzerland, 98% purity) was added in amounts adjusted to the glucose content to ensure comparable sweetness and maintain blinding across all interventions. All interventions were as sweet as the intervention with the highest glucose content (intervention 3 [I3]). In place of C8, the control intervention contained a high oleic sunflower oil (Michael Hinterauer, Stübener Kräutergarten, Germany; 87% oleic acid, 4.3% linoleic acid, 3.7% palmitic acid, 3.1% stearic acid) and no glucose. Sunflower lecithin (Ivovital, Germany, ≥ 95% purity) was added to the control intervention to create an emulsion. All intervention drinks were emulsified for 70 s using a high-performance blender with 1,200 watts (Ninja BN750EU, UK). Table 1 presents the precise compositions of the interventions in g/kg bw (bodyweight). The dosages of C8 and glucose were determined on the basis of the results of a meta-analysis of the influence of glucose and C8 on ketone body synthesis^27^. Interventions 1–3 (I1–I3) investigated the cut-off of C8-induced ketone body synthesis through glucose intake, with constant amounts of C8 and increasing amounts of glucose between interventions. Linearity was tested using I1, I4 and I5 by increasing the amounts of glucose and C8 in parallel between interventions.

**Table 1:** Composition of the six interventions including control.

Abbreviation	Dosage in Oil	Dosage in Glucose	Water	Aroma	Sweetener
I1)	0.2 g/kg bw C8	0.2 g/kg bw	250 ml	different flavours, 0.15 µl	Sucralose ^a^
I2)	0.2 g/kg bw C8	0.4 g/kg bw	250 ml	different flavours, 0.15 µl	Sucralose ^a^
I3)	0.2 g/kg bw C8	0.6 g/kg bw	250 ml	different flavours, 0.15 µl	–
I4)	0.3 g/kg bw C8	0.3 g/kg bw	250 ml	different flavours, 0.15 µl	Sucralose ^a^
I5)	0.4 g/kg bw C8	0.4 g/kg bw	250 ml	different flavours, 0.15 µl	Sucralose ^a^
C)	0.2 g/kg bw sunflower oil	–	250 ml	different flavours, 0.15 µl	Sucralose ^a^

^a^Dosage depending on the amount of glucose. All interventions correspond to the sweetness of the intervention with 0.6 g/kg bw glucose (I3). bw = body weight.

### Experimental design

**Fig. 1:**
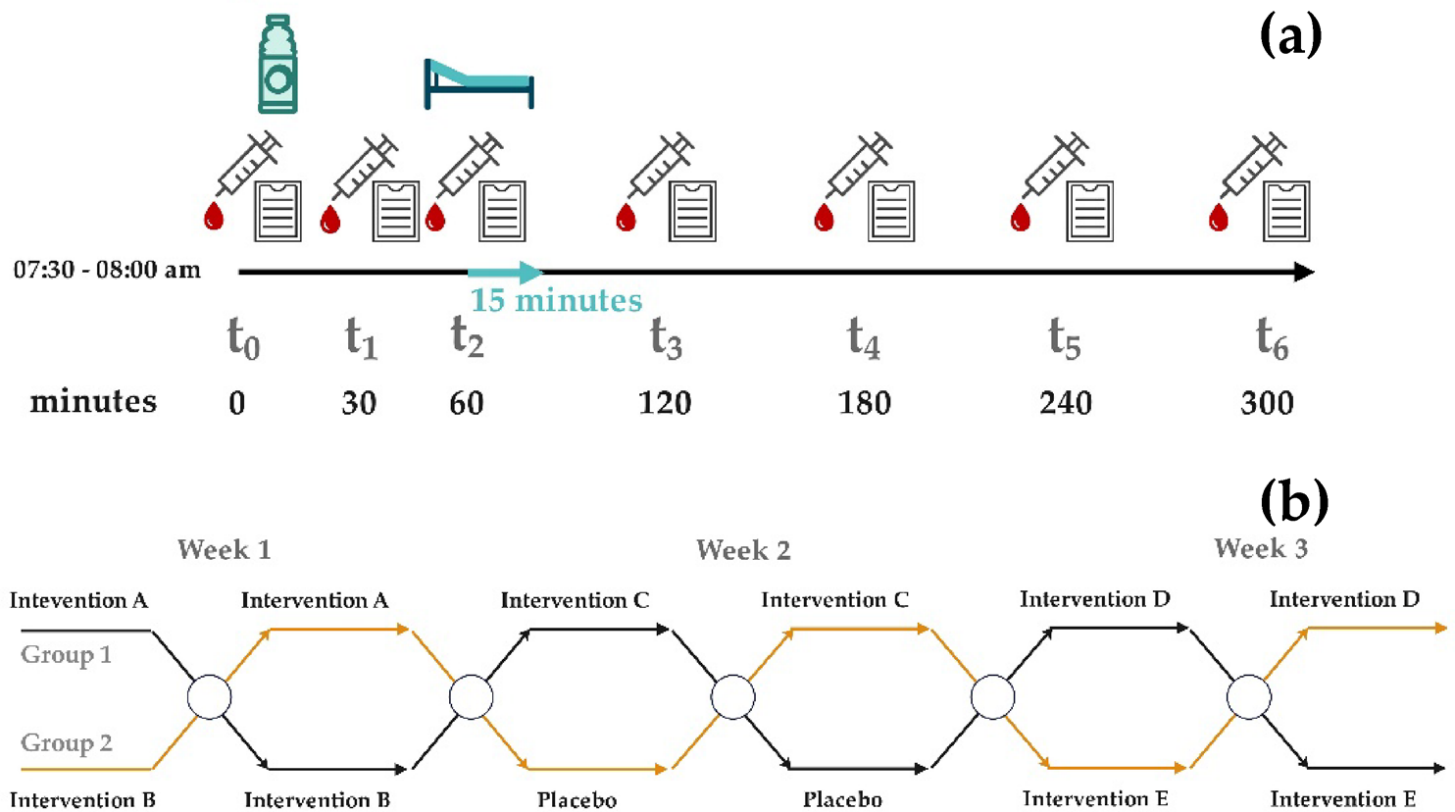
Description of the study design; (**a**) Measurement points and intervention procedure (bottle = intervention; syringe = blood sample; paper = documentation of side effects; examination bed = indirect calorimetry); (**b**) Schematic of randomized intervention sequence in two groups. Intervention labels (A–E, placebo) are shown for illustrative purposes and do not reflect intervention nomenclature.

This was a monocentric, randomised, controlled, double-blind study with a crossover design. The participants arrived at the study centre in the morning between 7:30 and 8:00 a.m. after fasting for at least 12 h overnight (see Fig. 1a). The consumption of alcohol and cannabis was prohibited on the days prior to the study. The participants were randomly allocated to one of two groups in advance (intervention-order randomization) and received interventions in different orders (see Fig. 1b). The six study days were spread over three weeks per participant, with two non-consecutive study days per week, each following the same procedure. On the study days, an intravenous catheter was inserted into the forearm in the morning, through which seven blood samples were obtained. The intervals between the blood samples are illustrated in Fig. 1a. Following the collection of the initial blood sample (t_0_), the participants ingested one of the six intervention drinks, which were contained in opaque cups with lids and were mixed immediately prior to consumption. The supervising staff and the participants were blinded to the intervention. The drink was consumed in its entirety within a span of 10 min. The order of the interventions differed between the groups (see Fig. 1b). There were no differences in taste between the interventions. Following the intervention, participants were permitted to consume water and caffeine- and sugar-free tea exclusively. The participants were instructed to refrain from strenuous physical activity. Side effects were documented in a logbook at each blood sample collection. At t_0_ and 60 min after the intervention (t_2_), indirect calorimetry (Q-NRG, Cosmed, Germany) was additionally performed as a secondary, explorative outcome using the canopy method. Outcomes included respiratory quotient (RQ) and the relative contribution of fat, carbohydrate, and protein to total energy expenditure. Due to logistical constraints and measurement duration, it was not feasible to perform indirect calorimetry in all participants on each study day. Indirect calorimetry was performed in those participants for whom the predefined time window allowed assessment without interfering with the primary study procedures. The time point t_2_ was selected on the basis that MCT induces ketone body synthesis within 60 min^27^. It was therefore anticipated that the metabolisation of MCT would be evident at this time point. Prior to the measurement, the room was ventilated and the participants were instructed to remain motionless for approximately 10 min. This was followed by a 15-minute measurement of the participant in a supine position. The ‘best five minutes’ were automatically selected by the device software in accordance with the manufacturer’s recommendation^31^. This study was conducted in accordance with the Consolidated Standards of Reporting Trials (CONSORT) guidelines^32^.

### Plasma metabolite analyses/laboratory analyses

Blood samples were centrifuged at 10 °C for 10 min, and plasma was subsequently stored at -80 °C for up to five months before analysis. The measured metabolites (glucose^33^, insulin^34^ and βHB ^35,36^) are known to be stable at this temperature. Glucose, insulin and βHB were determined using standard procedures at the university hospital laboratory Muenster, Germany. Plasma glucose concentrations were assessed enzymatically with the hexokinase method on an automated chemistry analyzer (Cobas C702, Roche Diagnostics GmbH, Mannheim, Germany). Plasma insulin was quantified by electrochemiluminescence immunoassay on a Cobas e801 analyzer (Roche Diagnostics GmbH, Mannheim, Germany). βHB was measured enzymatically using a kit from Randox Laboratories Ltd. (Crumlin, United Kingdom) on a Cobas Mira Plus analyzer (Roche Diagnostics GmbH, Mannheim, Germany).

### Statistical analyses

For βHB and insulin, the area under the curve (AUC) was calculated using the trapezoid method^37^ with Microsoft Excel (version 2108). Statistical analyses were performed using SPSS Statistics software (version 29.0.1.0, IBM Corp., Armonk, NY; USA). The Shapiro-Wilk test and histograms were used to determine that plasma metabolite data were not normally distributed. Consequently, Friedman tests with accompanying pairwise post-hoc analysis with Bonferroni correction in the event of a significant difference were performed for the parameters βHB, glucose and insulin in serum to test for differences between interventions at each time point (t_0_–t_6_). The AUC of βHB and insulin and the effect of treatment – defined as the difference between the maximal βHB value of each intervention and the corresponding control value – were analyzed accordingly. Wilcoxon’s Signed Rank test was used to assess changes over time relative to the baseline of βHB, by comparing each time point to t_0_ within each intervention. To investigate the relationship between AUC (βHB) and C8/glucose dosage, both Pearson and Spearman correlation analyses (two-tailed) were used. The strength of the correlations was interpreted according to Cohen (1988): |r| = 0.10–0.29 = weak; |r| = 0.30–0.49 = moderate; |r| ≥ 0.50 = strong^38^. Since indirect calorimetry was performed on selected participants at random and therefore no complete paired sample was available, the Kruskal-Wallis test was used to analyse the differences in macronutrient-specific energy expenditures. For the calculation of the sample size, the following values were assumed: 0.6 mmol/L (± 0.1 mmol/L) for I1^12,29^; 0.39 mmol/L (± 0.084 mmol/L) for I2^27^; and 0.2 mmol/L for I3. Based on these assumptions, a sample size calculation was carried out for a three-group ANOVA with an assumed correlation of ~ *r* = 0.5 (simulation program: R). The relevant statistical test was the F-test *p*-value of the treatment factor (2 degrees of freedom). A sample size of *n* = 10 with a power of 97.2% (requirement > 80%) was determined. Including dropouts (30%), the calculated total number of subjects was *N* = 13. The significance level was set at α = 0.05 for all statistical analyses.

## Results

Of the 13 participants initially enrolled, four withdrew for personal reasons (*n* = 1) or due to blood sampling (*n* = 3). Two additional participants were subsequently enrolled to maintain the planned sample size. Eleven healthy participants (11 women, 0 men) with an average age of 22.5 ± 1.9 years and a BMI of 20.9 ± 2.3 completed the study. The baseline characteristics are shown in Table 2. In the interventions to determine the cut-off point (I1–I3), 12.0 ± 1.6 g C8 and 12.0 ± 1.6 g glucose (I1), 23.9 ± 3.2 g glucose (I2) and 35.9 ± 4.9 g glucose (I3) were administered. To test for potential linearity, in addition to I1, 17.9 ± 2.4 g C8 and glucose were consumed during I4, and 23.9 g ± 3.2 g C8 and glucose were consumed during I5. C contained 12.0 ± 1.6 g sunflower oil.

**Table 2:** Baseline demographic, anthropometric and biochemical characteristics of the participants.

Characteristics	Value
Sex (female / male / all genders)	11 (11/0/0)
Age (years)	22.5 ± 1.9 ^a^
BMI (kg/m^2^)	20.9 ± 2.3 ^a^
Weight (kg)	59.8 ± 8.1 ^a^
Height (cm)	169.2 ± 4.9 ^a^
Plasma measurements (venous) ^b^	
Fasting βHB (mmol/L)	0.09 ± 0,03 ^a^
Fasting glucose (mg/dL)	86.4 ± 0.5 ^a^
Fasting insulin (µU/mL)	8.6 ± 1.2 ^a^
Indirect calorimetry	
Respiratory quotient (RQ)	0.79 ± 0.06 ^a^
Fat (%) ^c^	73.4 ± 21.9 ^a^
Carbohydrate (%) ^c^	26.6 ± 21.9 ^a^
Protein (%) ^c^	0.0 ± 0.0 ^a^

^a^ Mean values and standard deviations.

^b^ baseline plasma concentrations were calculated as the mean of t_0_ values across all six intervention days.

^c^ fat/carbohydrate/protein percentage of energy expenditure.

### Beta-hydroxybutyrate and AUC analysis of cut-off interventions

**Fig. 2:**
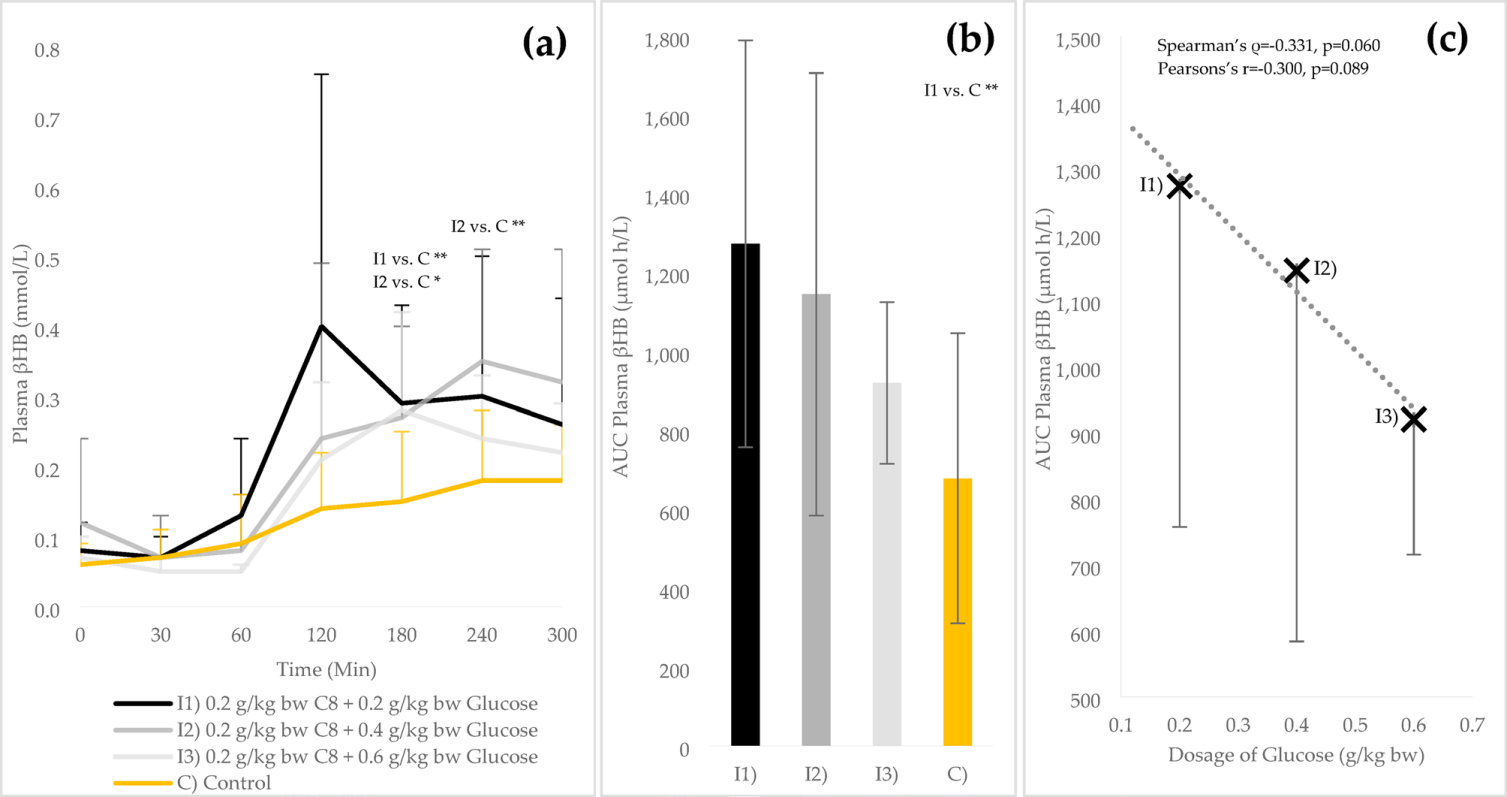
Plasma βHB levels (mean values ± standard deviation) during the cut-off interventions and control: (**a**) Absolute values of βHB over time (mmol/L); (**b**) Area under the curve of βHB (AUC; t_0_ – t_6_; µmol h/L); (**c**) AUC of βHB (I1, I2 and I3) in relation to glucose intake, including linear regression line. bw = body weigth. *Significant difference between the different test conditions (*p* ≤ 0.05); ** (*p* ≤ 0.01).

After 60 min (t_2_), the mean βHB values of all cut-off interventions (I1, I2 and I3) increased (see Fig. 2a). After 120 min, βHB levels were 67–90% higher in I1 compared to I2 and I3, with no significant differences between interventions (*p*=0.173). Thereafter, the curves converged. The βHB increase of all interventions, including C, was significant from t_3_ compared to t_0_ (*p*≤0.05). Compared to C, I1 was significantly increased at t_4_ (0.29 ± 0.14 mmol/L vs. 0.15 ± 0.10 mmol/L; *p*=0.010), and I2 was significantly increased at both t_4_ (0.27 ± 0.13 mmol/L vs. 0.15 ± 0.10 mmol/L; *p*=0.039) and t_5_ (0.35 ± 0.16 vs. 0.18 ± 0.10; *p*=0.010). While I1 reached a maximum value of 0.40 ± 0.11 mmol/L at t_3_, the βHB concentration for I2 was highest at t_5_ (0.35 ± 0.16 mmol/L) and at t_4_ for I3 (0.28 ± 0.14 mmol/L). The mean AUC for βHB was reduced by 10.1% and 27.7% at I2 and I3 compared to I1 (*p*>0.05; see Figure 2b), with the AUC of I1 significantly increased compared to C (1273 ± 516 µmol h/L vs. 678 ± 368 µmol h/L, *p*=0.008). A moderate, non-significant negative correlation was observed between AUC and the glucose dosage, regardless of the test method (Spearman’s ρ=-0.331, *p*=0.060; Pearson’s *r*=-0.300, *p*=0.089; see Figure 2c).

### Beta-hydroxybutyrate and AUC analysis of linearity interventions

**Fig. 3:**
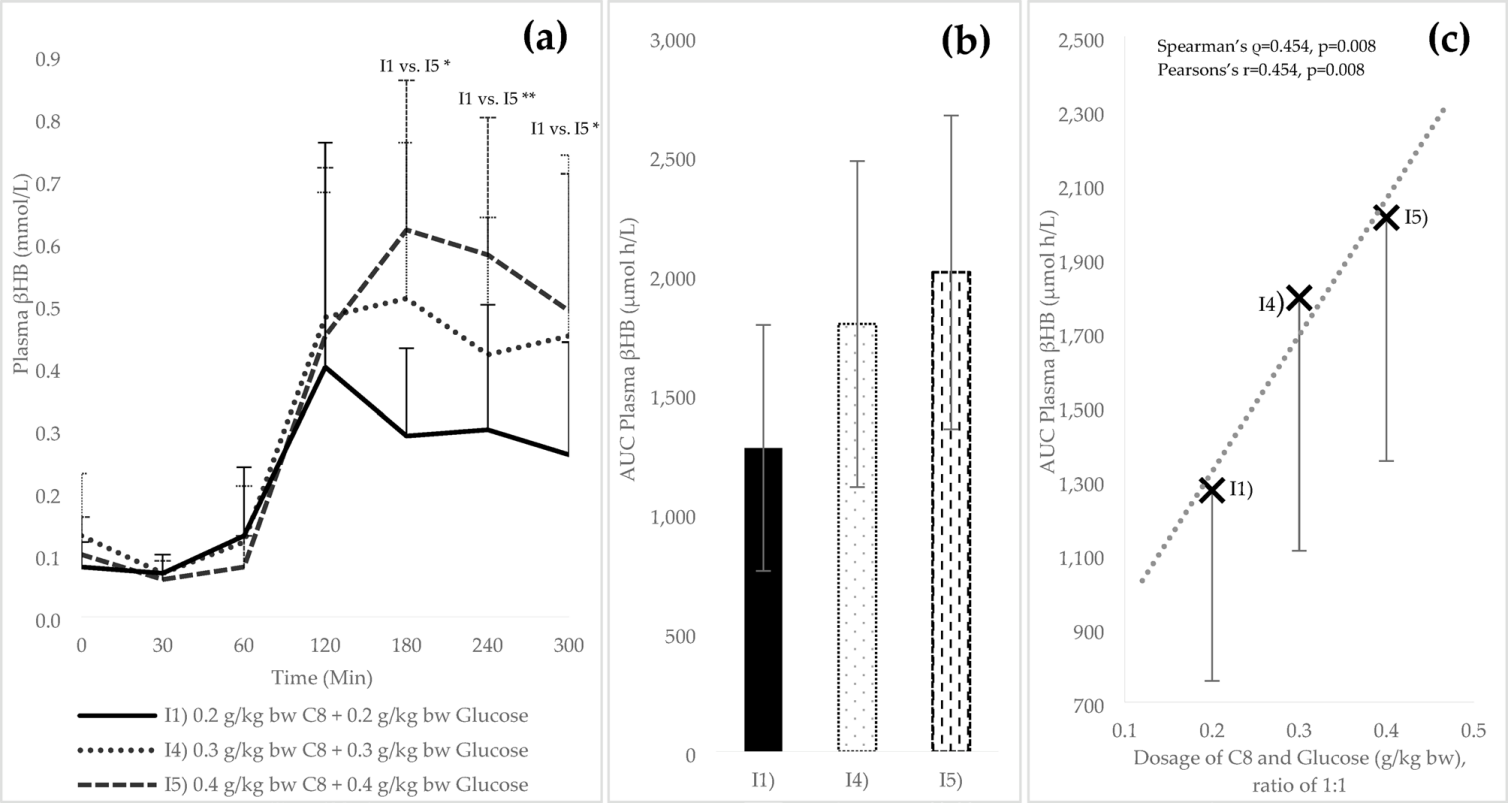
Plasma βHB levels (mean values ± standard deviation) during the linearity interventions: (**a**) Absolute valuesof βHB over time (mmol/L); (**b**) Area under the curve of βHB (AUC; t_0_ – t_6_; μmol h/L); (**c**) AUC of βHB (I1, I4 and I5)in relation to glucose/C8 intake, including linear regression line. bw = body weigth. *Significant difference between the different test conditions (*p* ≤ 0.05); **(*p* ≤ 0.01).

In addition to I1, the mean βHB values of the other linearity interventions I4 and I5 also increased after 60 min (t_2_) (see Fig. 3a). I1 reached its maximum after 120 min (0.40 ± 0.36 mmol/L), I4 (0.51 ± 0.25 mmol/L) and I5 (0.62 ± *0.24* mmol/L) after 180 min. The graphs then declined slightly. The βHB increase in all linearity interventions was significant from t_3_ compared to t_0_ (*p* ≤ 0.05). I5 was significantly increased compared to I1 at the time points t_4_–t_6_ (t_4_: 0.62 ± 0.24 mmol/L vs. 0.29 ± 0.14 mmol/L; *p* = 0.017; t_5_: 0.58 ± 0.22 mmol/L vs. 0.30 ± 0.20 mmol/L; *p* = 0.009; t_6_: 0.49 ± 0.22 mmol/L vs. 0.26 ± 0.18 mmol/L; *p* = 0.032). The mean AUC for βHB of I4 and I5 was increased by 40.8% and 57.9% compared to the AUC of I1 (see Fig. 3b). A moderate positive correlation was observed between the AUC and the C8 dosage (Spearman’s ρ = 0.454, *p* = 0.008; Pearson’s *r* = 0.454, *p* = 0.008; see Fig. 3c). The βHB curves and AUCs for all interventions, including cut-off and linearity interventions and control, are shown in Supplementary Materials, Figure S1.

### Glucose and insulin responses

**Fig. 4:**
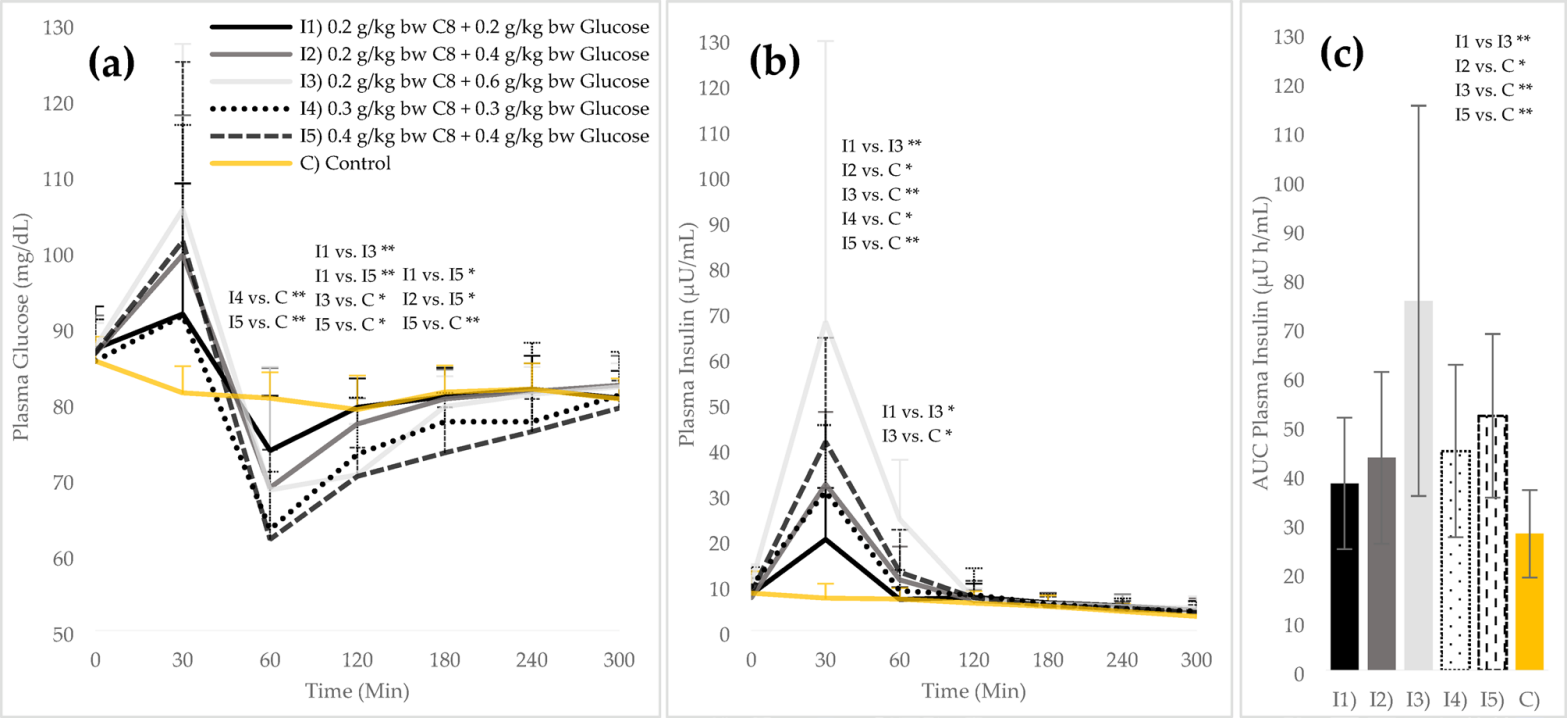
Plasma glucose and insulin levels (mean values ± standard deviation) during the intervention days: (**a**) Plasma Glucose, absolute values (mg/dL); (**b**) Plasma Insulin, absolute values (μU/mL); (**c**) AUC of Insulin (t_0_ – t_6_; μU h/mL). bw = body weight. *Significant difference between the different test conditions (*p* ≤ 0.05); ** (*p* ≤ 0.01)

After 30 min (t_1_), plasma glucose reached a maximum value in all interventions except C (see Fig. 4a). The values ranged from 91.5 ± 25.1 (I1) to 105.5 ± 21.8 mg/dL (I3). Interventions with higher glucose amounts achieved higher plasma glucose levels, but there were no significant differences between the interventions at t_1_. Plasma insulin also reached a maximum value at t_1_ in all interventions except the control, up to 67.8 ± 61.6 µU/mL (I3), with all interventions except I1 significantly increased compared to C (*p* = 0.021/0.000/0.021/0.000; I2/I3/I4/I5) (see Fig. 4b). The insulin concentration of I5 (41.4 ± 22.9 µU/mL) at t_1_ was 29.0% higher than that of intervention I2 (32.1 ± 15.9 µU/mL) with the same amount of glucose but a higher proportion of C8 of 0.4 g/kg bw (*p* = 1.000). The AUC of I5 was also 14.5% higher than that of I2 (*p* = 1.000; see Fig. 4c). At t_2_, plasma glucose and insulin decreased in all interventions except C, and plasma glucose reached the respective minimum (62.0 ± 11.8 [I5] – 73.7 ± 7.2 mg/dL [I1]). With the same glucose intake (0.4 g/kg bw), I5 with 0.4 g/kg bw C8 had a greater drop in blood glucose at t_2_ than I2 with 0.2 g/kg bw C8 (63.3 ± 7.6 vs. 68.8 ± 15.8 mg/dL; *p* = 1.000). The interventions for testing linearity (I1, I4, I5) showed that I4 and I5 with higher C8 amounts led to a greater drop in glucose than I1 (63.3 ± 7.6 [I4] vs. 73.7 ± 7.2 [I1]; *p* = 0.340 / 62.0 ± 11.8 [I5] vs. 73.7 ± 7.2 [I1]; *p* = 1.000). By t_6_, the glucose curves were approaching the initial level.

### Macronutrient distribution of energy expenditure

**Fig. 5:**
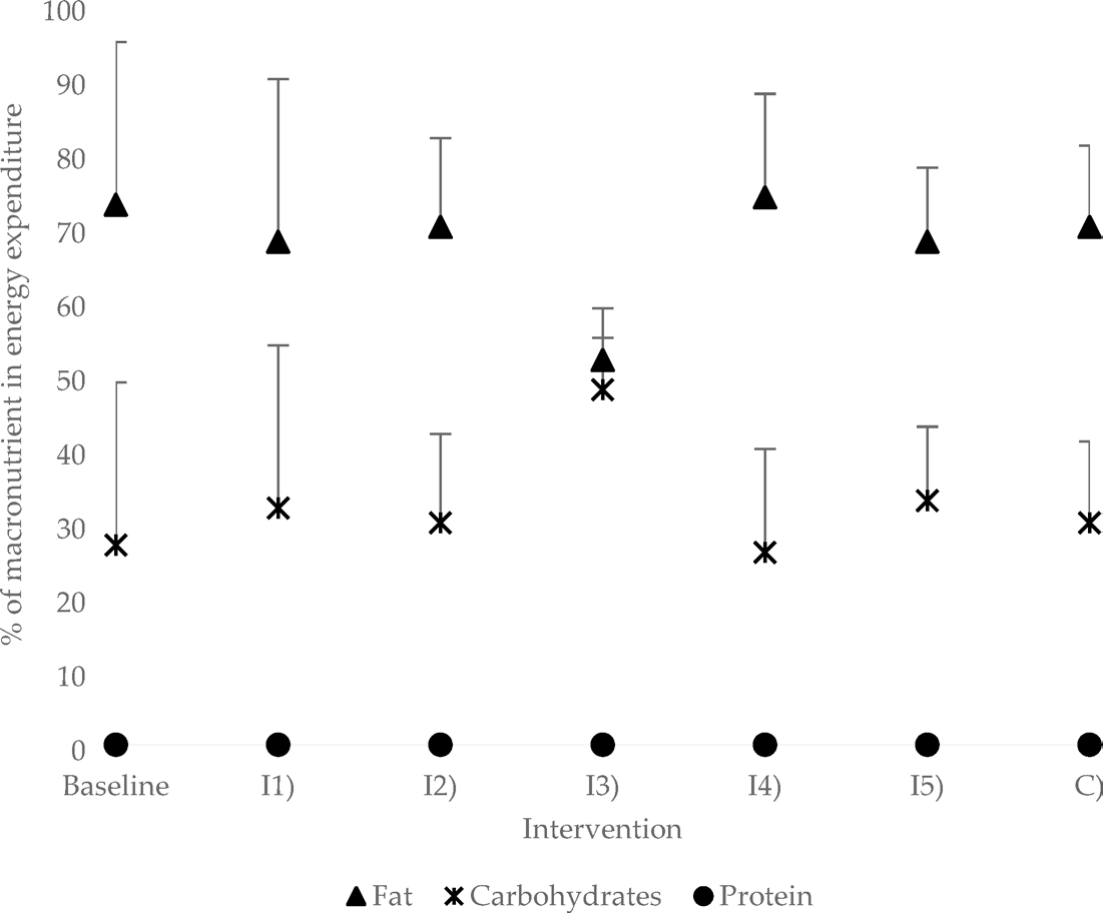
Distribution of macronutrients for energy expenditure at time t_2_ and baseline (mean values ± standard deviation; N Baseline = 11; N I1) = 7; N I2) = 7; N I3) = 5; N I4) = 5; N I5) = 6; N C) = 7).

In all interventions and at t_0_, the average fat content of energy consumption was 68–74 ± 10–22%. At the highest glucose dosage of 0.6 g/kg bw and intake of 0.2 g/kg bw C8 (C), the proportion fell to 52 ± 7%. The mean CH percentage of energy consumption was 26–33 ± 10–22% for all interventions and at t_0_. The highest glucose dose administered resulted in an increase in CH utilisation of 48 ± 7%. The mean protein percentage of energy consumption was found to be 0 ± 0% for all measurements (see Fig. 5). The respiratory quotient (RQ) exhibited a range of 0.78–0.80 ± 0.03–0.07 for all interventions and t_0_, while the value for the highest glucose dosage was 0.85 ± 0.02. The differences were not statistically significant.

### Side effects

**Fig. 6:**
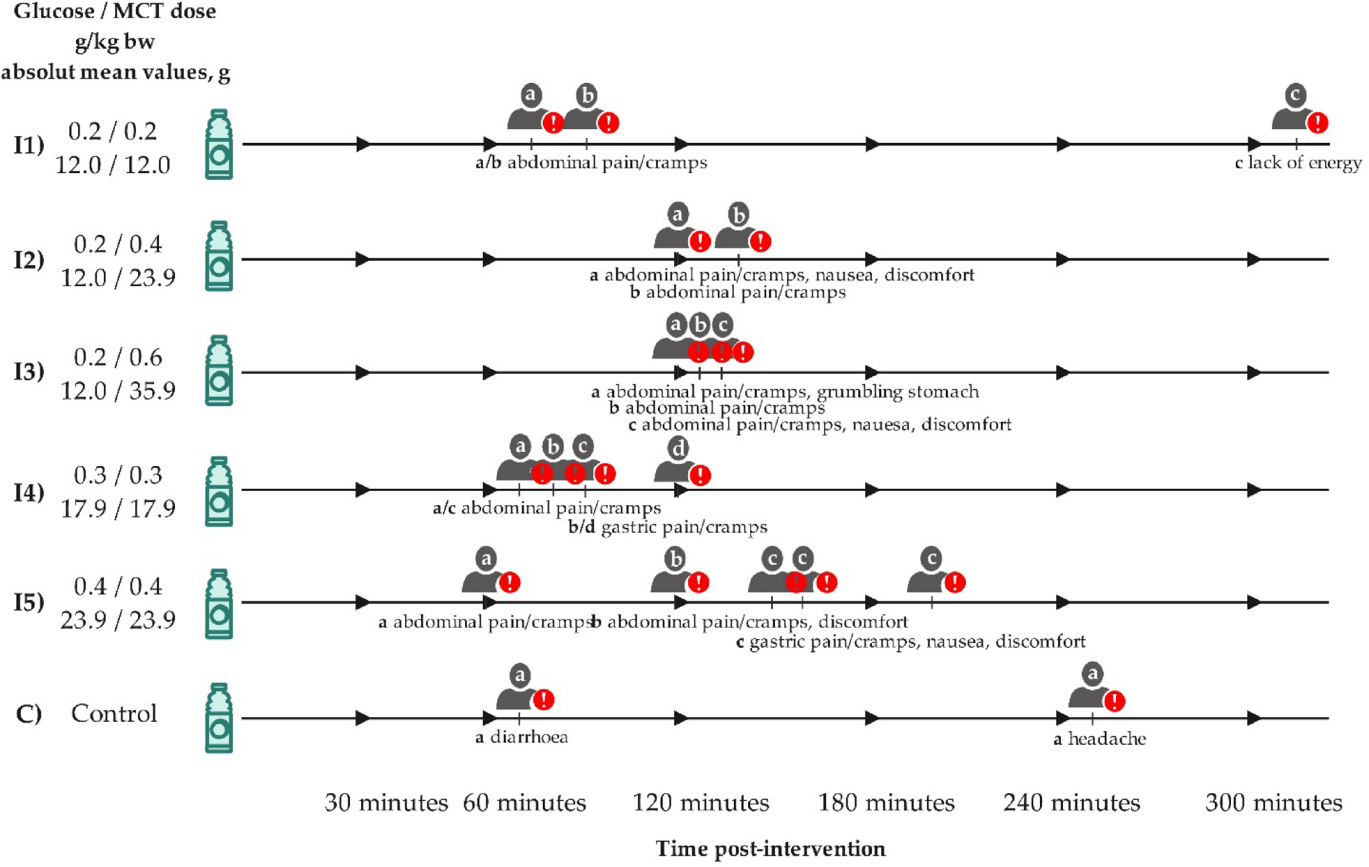
Time course and nature of side effects during the interventions (*N* = 11). The participant letters are not contiguous across inventions. The letters show the course of side effects in individual participants (a = one participant, b = another participant etc.).

Mild to moderate side effects occurred in all interventions (see Fig. 6). At C8 doses of 0.2 g/kg bw (⌀ 12.0 g, I1–I3), 2–3 participants (18.2–27.3%) reported abdominal pain and cramps, nausea, discomfort and lack of energy at one point in time. At 0.3 g/kg bw (⌀ 17.9 g, I4) C8, 4 participants (36.4%) reported abdominal and gastric pain and cramps on a single occasion. At the maximum C8 dose of 0.4 g/kg bw (⌀ 23.9 g, I5), 3 participants (27.3%) reported the side effects already mentioned, with one participant also reporting nausea. One participant suffered from repeated side effects. During the control intervention, one participant reported diarrhea and headaches. Across all interventions, the most common side effects were abdominal pain/cramps (11x) and general discomfort (4x). The occurrence of side effects was recorded at the earliest 60 min following intervention intake, with 63.2% of cases manifesting after 120 min or later. The duration of the side effects ranged from 15 to 120 min.

## Discussion

This study aimed to determine the cut-off point for ketone body synthesis at a constant C8 level and increasing glucose levels. Secondary objectives included examining the influence of linearly increasing amounts of C8 and glucose on the formation of βHB.

In 2016, Cunnane et al. suggested a linear relationship between an MCT dosage of up to 70 g and plasma βHB concentration based on data of existing studies. However, this assumption did not consider the participants’ health status, the composition of the MCT including C8 content, the preceding fasting period or the accompanying food intake^39^. Accordingly, Lin et al. (2021) questioned the existence of a linear relationship between MCT intake and ketone body production^40^. A meta-analysis of seven studies involving C8 and carbohydrate-containing interventions revealed that higher amounts of C8 did not necessarily lead to higher ketone levels when CH were consumed in parallel. This suggests that the influence of increasing amounts of C8 on ketone body synthesis is non-linear. However, the review was limited by the heterogeneous intervention conditions of the included studies^27^. Linearly increasing amounts of C8 and glucose in a 1:1 ratio in the C8arbo I study showed an increase in ketone body synthesis (t_max_) as well as an increase in the AUC (βHB), with a moderate positive correlation between the AUC and C8 dosage. Currently, due to the limited available data, it is unclear whether this effect will persist with a further linear increase in C8 and glucose dosages above 0.4 g/kg bw, or if it applies equally to interventions without accompanying glucose intake.

A meta-analysis showed that total ketones after intake of C8 and CH in a ratio of 1:2.9 (18.6 ± 0.9 g C8) and 1:5.4 (11.4 ± 1.0 g C8) over 4 h ranged from 0.2 to 0.38 mmol/L^27^. At comparable C8 dosages, the C8arbo I intervention I5 in a 1:1 ratio resulted in a higher t_max_, while I3 in a 1:3 ratio was also inhibited at t_max_. The comparison therefore suggests that a C8:CH ratio of 1:2.9 or 1:3 (C8arbo I study) or higher appears to largely mask the ketogenic effect of MCT. In comparison, increased ketone body synthesis appears possible in a 1:1 ratio. Based on the available data, a C8:CH ratio of 1:2 appears to inhibit ketone body synthesis to a limited extent within five hours, a finding that is also supported by a study by Norgren et al. (2020) involving a C8:CH ratio of 1:2.5 (20 g C8)^11^. In a study conducted by van Wymelbeke et al. (1998), the comparatively high intake of 43 g MCT (C8 and C10; specifics not provided) and 84.5 g CH (starchy pasta, tomato sauce) with an MCT: CH ratio of 1:2 led to a stable increase in ketone bodies to > 0.3 mmol/L ^41^. This is comparable to the C8arbo I intervention I2 in a C8:CH ratio of 1:2, which contains approximately 3.5 times less CH and MCT than the intervention by van Wymelbeke et al. (1998). There are indications that an MCT:CH ratio of 1:2 could also be applied to increased CH amounts above the CH intake limit of 50 g usually specified for ketogenic diets or very low-carbohydrate diets^42,43^ with correspondingly increased MCT amounts without reduced βHB increase. However, βHB rose immediately after intake of the starch-containing intervention^41^, whereas there was a 60-minute lag time in the glucose-containing interventions. In a study by Kanta et al. (2025), a maximum value of 0.7 ± 0.1 mmol/L βHB was reached after 90 min, falling slightly up to five hours after ingestion of 35 g of C8 (99% C8) and 9.6 g of CH (mainly lactose and some sucrose; C8:CH ratio of 1:0.27)^44^. By contrast, the βHB curve of I5 (24 g C8; 1:1) was only slightly lower. Thus, an MCT intake with a C8:CH ratio of 1:1 appears to largely mask the anti-ketogenic effect of glucose. The significantly increased βHB values of the C8arbo I interventions I1 (ratio 1:1) and I2 (ratio 1:2) at t_4_ and t_5_ compared to C – in contrast to the glucose-richest intervention I3 (ratio 1:3) – as well as the AUC (βHB) comparison with low AUC of intervention I3 and significantly increased AUC of I1 compared to C confirm the correlations presented. Only at I3 did the RQ value increase, indicating a higher proportion of CH in energy consumption and reduced fat utilisation. In a randomised crossover study with healthy men, a high-carbohydrate meal led to significantly higher glucose oxidation than a high-fat meal (1.007 ± 0.192 vs. 0.473 ± 0.177 mg/kg/min; *p* = 0.07). Lipid oxidation was higher after a normal carbohydrate-rich meal at the subsequent time points (120–240 min) than after the carbohydrate-rich meal, with a significantly higher AUC of the fat oxidation curve (234 ± 13 vs. 185 ± 18; *p* = 0.03)^45^. These results highlight the meaning of the increased RQ value at the highest glucose intake (I3), while Frayn (1983) and Ferrannini (1988) demonstrated that changes in ketone and lactate metabolism may interference the interpretation of indirect calorimetry, as ketone body and lactate metabolism contribute to O_2_ and CO_2_ exchange beyond pure substrate oxidation^46,47^. This study suggests a linear relationship between increasing glucose levels and decreasing βHB levels. It is therefore currently unclear whether the 1:3 ratio is a cut-off point or whether CH intake becomes too low above a certain range due to negative linearity. Doubling and tripling the glucose intake almost halved βHB levels after 120 min, meaning that the increasing glucose intake represents a substantial inhibitory factor on ketone body synthesis.

Glucose and insulin initially showed a dose-dependent relation. The transient drop below baseline at t_2_ likely reflects the bolus-like, pulsatile insulin secretion^48,49^, followed by stabilization. This pattern indicates endogenous glucose production after overnight fasting, likely through gluconeogenesis^11,12,50,51^ and/or glycogenolysis^52^. In comparison with Eckstein et al. (2021) after pure glucose intake of 1 g/kg bw glucose without MCT as well as Matsuda et al. (1999) using an oral glucose tolerance test, a rise in blood sugar was observed, stabilising in the range of approximately 30 to 50 min after intervention intake and falling steadily to the baseline value after approximately 120 min^53,54^. In contrast to the C8arbo I results, an immediate, sharp drop in blood sugar after a peak, including subsequent baseline approximation, was not observed in the studies mentioned. This suggests, that MCT intake may be relevant to this mechanism. The literature describes a delayed and inhibited MCT-induced βHB increase with parallel glucose intake^11,12^. While in the study by Heidt et al., a clear MCT-induced βHB increase also occurred after 30–60 min at 0.2 g/kg bw (approx. 12.8 g) glucose^12^, Norgren et al. showed an MCT-induced βHB increase after only 30 min with parallel intake of 50 g glucose^11^. The observed lag time does not occur after pure MCT intake^12^. If glucose is ingested at the same time, it can be assumed that both glucose and the MCFA derived from MCT are initially oxidised via acetyl-CoA in the citric acid cycle^55,56^. As glucose availability and oxidation decrease, the acetyl-CoA formed may preferentially be available for ketone body synthesis^57^, which could explain the increase after 60 min. In addition, comparatively high insulin levels were achieved in C8arbo I at t_1_, and it was found that higher MCT amounts, even with the same glucose amounts, led to greater drops in blood sugar (I2 vs. I5; I1 vs. I4/I5). The relationship between ketone body synthesis and insulin is described in the literature as fundamentally close and counteractive^58^. The insulin level increased significantly in all C8arbo I interventions except C at t_1_ to 20.0 ± 11.3 (I1) to 67.8 ± 61.6 µU/mL (I3), and βHB increased only after insulin levels had markedly declined after 60 min. In line with the present findings, a study by Heidt et al. (2021) showed that insulin levels after MCT supplementation alone (0.5 g/kg bw) rose from 4.3 (2.3–6) µU/ml to 5.1 (1.9–6.7) µU/ml (median value [IQR]) after 30 min, which was non-significant higher than the control value (water) (*p* = 0.8)^12^. The present effect, whereby MCT intake leads to increased insulin levels (I2 vs. I5), has also been observed in four other human intervention studies^44,59–61^ and one study on rats^62^. In particular, Kanta et al. (2025) observed an MCT-induced increase in insulin after administering MCT and CH in simultaneously^44^, as in C8arbo I. The underlying mechanism of MCT-enhanced insulin secretion remains unclear^63^. In animal models C8:0 and C10:0 have been shown to enhance glucose-induced insulin secretion via incretin hormones like GLP-1 and calcium-dependent signaling pathways^63–66^. However, evidence in humans remains limited, and an additional insulin-independent regulatory mechanism may help prevent excessive ketone body accumulation^67,68^.

MCT are associated with various dose-dependent side effects [21]. Abdominal cramps and diarrhea are particularly relevant, as demonstrated by a study in which all six participants experienced these side effects when given a pure intake of approximately 40 g MCT (C8/C10; 60 mmol)^69^. Huttenlocher et al. (1971) also reported gastrointestinal discomfort after ingesting 39 ml of MCT, which was severely reduced by emulsification and slow consumption of the MCT drink^70^. Courchesne-Loyer et al. (2017) confirmed the increase in tolerability through emulsification^29^. Although side effects occurred, the interventions were well tolerated overall.

All participants were female despite recruitment being open to all sexes. Women in the follicular phase show lower fasting insulin levels and higher insulin sensitivity than men, along with a greater reliance on lipid oxidation at rest^71^. Although evidence on sex-specific substrate metabolism is mixed^71^, several studies report higher ketone body concentrations in women during fasting, with differences most pronounced within the first 72 h^72–74^. A study by Marinou et al. (2011) demonstrated that women converted more circulating fatty acids into ketone bodies in the liver^74^. These results indicate sex-specific differences in hepatic fatty acid utilisation and ketone body synthesis. Bradshaw et al. (2024) also report that women have lower intra-individual variability than men in relevant fasting metabolic parameters (including plasma glucose, insulin, resting metabolic rate)^75^. Given the small sample size, the findings should be interpreted with caution and may not generalize to men, older populations, or individuals with metabolic disorders.

## Conclusions

This study compared interventions with different dosages of glucose at uniform C8 amounts and interventions with parallel increases in C8 and glucose amounts in terms of their effects on ketone body synthesis. In summary, it was found that interventions with low (0.2 g/kg bw) and medium glucose levels (0.4 g/kg bw) achieved significant βHB increases at some time points and AUC compared to the control group, in contrast to the intervention with high glucose levels (0.6 g/kg bw). In addition, a moderate positive correlation between the AUC of βHB and C8 dosage and corresponding linearity was observed with parallel C8 and glucose increases in a 1:1 ratio without any signs of disruption to ketone body synthesis. A moderate negative, non-significant correlation was also shown with increasing glucose dosages at a constant C8-intake. This suggests that ketone body synthesis tends to decrease with increasing glucose levels. The C8:glucose ratio of 1:1 to 1:2 indicates that ketone body synthesis remains fairly stable after a 60-minute lag time. A ratio of 1:3 shows a reduced increase in ketone bodies. It remains unclear whether the 1:3 ratio is a cut-off point or CH intake becomes too high above a certain range due to linearity. Furthermore, C8 caused a slight increase in insulin. The mechanisms by which MCT induce an increase in insulin are unclear and require further research. Emulsified intervention drinks containing 0.2–0.4 g/kg bw of C8 exhibited mild to moderate gastrointestinal side effects.

## Supplementary Information

Below is the link to the electronic supplementary material.

Supplementary Material 1

## Data Availability

The datasets used and/or analysed during the current study are available from the corresponding author on reasonable request.
