# Supplementary material for: Effect of glucose on medium chain triglyceride induced ketosis in healthy adults in a randomized, double-blind, controlled study
Source: Sci Rep. 2026 Apr 9;16:12049. doi: 10.1038/s41598-026-47702-4 (PMC13068931; doi:10.1038/s41598-026-47702-4)
Supplement: Supplementary file 1 — Supplementary Material 1 [file 41598_2026_47702_MOESM1_ESM.docx]

**Figure S1.** Plasma βHB levels over time (mmol/L) and area under the curve (AUC; t_0_ – t_6_; µmol h/L) during the cut-off and linearity interventions and control.
